# Effects of feces storage conditions for host-microbiota screenings in *C. elegans*

**DOI:** 10.3389/frmbi.2024.1426254

**Published:** 2024-12-19

**Authors:** Laury Caron, Claudia Miriam Alonzo De la Rosa, Khoudia Diop, Stéphanie Miard, Stefan Taubert, André Marette, Frédéric Picard

**Affiliations:** ^1^ Centre de recherche de l’Institut universitaire de cardiologie et de pneumologie de Québec, Québec, QC, Canada; ^2^ Faculty of Pharmacy, Université Laval, Québec, QC, Canada; ^3^ Institute of Nutrition and Functional Foods (INAF), Université Laval, Québec, QC, Canada; ^4^ British Columbia Children’s Hospital Research Institute, Vancouver, BC, Canada; ^5^ Centre for Molecular Medicine and Therapeutics, University of British Columbia, Vancouver, BC, Canada; ^6^ Department of Medical Genetics, University of British Columbia, Vancouver, BC, Canada; ^7^ Edwin S.H. Leong Centre for Healthy Aging, University of British Columbia, Vancouver, BC, Canada; ^8^ Department of Medicine, Faculty of Medicine, Université Laval, Québec, QC, Canada

**Keywords:** nematodes, *C. elegans*, mice, feces, microbiota, storage, freezing, host

## Abstract

**Background and aims:**

Current research on host-gut microbiota interactions is hindered by almost infinite bacterial combinations depending on intrinsic characteristics, environment, and health status, which prevents large-scale screenings in mammals. For these reasons, the bacterivore model organism *C. elegans* has been developed to test the effects of gut microbiota extracts from mammals. This study tested whether storage conditions of mouse feces and fecal extracts modify normal *C. elegans* healthspan.

**Methods:**

Feces from mice were processed for microbiota extraction after collection or after one or twelve months at -80 °C and compared to microbiota extracted six months before and left at room temperature. Extracts were probed for bacterial composition, viability, and nutritional content and tested in synchronized wild-type (strain N2) worms for food preferences and intake, development, fat accumulation, brood size, and maximal lifespan.

**Results:**

Long-term freezing of feces before microbiota extraction modified composition but did not negatively impact subsequent worm development, fat accumulation, reproduction, and maximal lifespan, whereas using samples extracted and left at room temperature after a long period of time resulted in robust avoidance and was detrimental for normal growth.

**Conclusions:**

Using frozen feces to test for impacts of microbiota in *C. elegans* appears an appropriate method since it did not affect normal biology and healthspan, which supports protocols with already existing feces stored in biobanks for high-throughput phenotype screenings.

## Introduction

Gut microbiota is now established as a powerful regulator of host biology (Lau et al., 2016; Vandeputte et al., 2017; Biagioli and Carino, 2019; Ghosh et al., 2022; Kieser et al., 2022; Vijay and Valdes, 2022), acting on key domains of health including energy metabolism, immune system (Kim et al., 2013), and behaviors (Forsythe et al., 2010; Carabotti et al., 2015). However, several effects of the gut microbiota on host physiology are still not fully understood. In mice, fecal microbiota transplantation is frequently employed as a mean to study the specific effects of certain types of gut microbiota (Chen et al., 2020; Fan and Pedersen, 2021; Parker et al., 2022), and complicated frameworks have been designed to progress into human studies (Cheng and Fischer, 2023). Studies have compared various conservation methods of microbiota, including freezing, to determine if feces of a healthy donor could be maintained in its original state (based on bacteria profiles) for a long period of time and for more than only one procedure (Papanicolas et al., 2019; Dorsaz et al., 2020).

Studies in invertebrate model organisms such as the bacterivore nematode worm *Caenorhabditis elegans* has now been developed to understand the complexity of microbiota-host interactions (Hsiao et al., 2013; Scott et al., 2017; Zhang et al., 2017; Kissoyan et al., 2022). *C. elegans* has been widely employed to study various *E. coli* strains (Khanna et al., 2016) and several other selected bacteria and fungi obtained from the human gut microbiota (MacNeil et al., 2013; Yilmaz and Walhout, 2014; Sim and Hibberd, 2016; Zhang et al., 2017). Moreover, based on its characteristics*, C. elegans* can serve as a valid model to rapidly screen for traits induced by microbiota extracted from fresh murine feces before embarking in more expensive studies in mammals. Indeed, we recently developed a protocol to extract and characterize the gut microbiota harvested from murine feces and to assess its influence on the physiology of *C. elegans* (Alonzo-De la Rosa et al., 2023). Compared to human feces, using murine feces allows for an easier control of genetics, health status, housing conditions, etc, isolating one experimental factor at a time. However, whether freezing of harvested murine feces affects viability, palatability, and subsequent biological processes in worms is still unknown. Answering this question could be highly valuable, as one could then use frozen fecal samples stored in biobanks instead of repeating protocols in mice to collect feces, reducing resources allocated to this task.

In this context, this study aimed at elucidating differences between feces stored at -80 °C for short or long periods and those freshly harvested by evaluating the energetic composition of the microbiota, its viability and bacterial composition, as well as its biological impacts on nematodes. Using our published approach and methodology, we compared microbiota extracted from feces of female mice aged six months and grouped according to their storage condition, being either frozen for more than a year, frozen for a month, harvested six months before and kept at room temperature (RT), and freshly harvested.

## Methods

### Feces harvest and storage conditions

Feces were harvested from six months old, C57BL/6J female mice (purchased from Jackson Labs, strain #000664) over a period of six weeks. All mice were housed in the same room under same conditions, with the same dark:light cycle and had access to regular chow diet (NIH-31 Teklad #7917, Envigo). All feces were harvested three hours following the opening of the lights according to our method previously described (Alonzo-De la Rosa et al., 2023). Mice were cared for and handled in accordance with the Canadian Guide for the Care and Use of Laboratory Animal and the Université Laval Institutional Animal Care Committee approved the protocol.

Upon harvest, half of the feces was put in 50 mL Falcon tubes and immediately stored at -80 °C and the other half was stored at room temperature in phosphate buffered saline (PBS). These were compared to feces that were already similarly stored at -80 °C for as much as one year and ready to be used and with feces that were stored at room temperature in PBS for 6 months (also from six months old, C57BL/6J female mice housed under the same conditions). Thereby, feces from different animals were pooled into four distinct groups according to the storage condition, namely those frozen for more than a year, those freshly harvested but frozen for a month, those freshly harvested and kept at room temperature, and those kept at room temperature for 6 months. This latter sample was tested as an extreme control to show the likely detrimental impacts of long-term RT-stored microbiota on worms.

The four pools of feces underwent the same homogenization to obtain microbiota extracts exactly as described (Alonzo-De la Rosa et al., 2023). Briefly, feces were placed in PBS (1 mL per 100 mg of feces), homogenized with robust agitation for 3 minutes, and then centrifuged at 800 g for 3 minutes. The supernatant was filtered with a 70 m pore size cell strainer to remove undesirable material, and then used for experiments described below.

### DNA extraction and quantification

Bacterial DNA was extracted and quantified as described (Alonzo-De la Rosa et al., 2023) using EtNa DNA extraction reagent (240 Mm NaOH, 74% ethanol, 2.7 mM EDA) and QIAmp mini spin column (QIAGEN) following manufacturer’s instructions. Bacterial DNA was quantified with a BioDrop analyser (Montreal Biotechnologies Inc., Canada). This quantification enabled the distribution of similar quantities of bacterial extracts (100 ng/uL) across the different groups throughout the experiments.

### 16S bacterial sequencing

Bacterial DNA was extracted from each sample by using the QIAamp PowerFecal Pro DNA kit (QIAGEN) and following manufacturer’s instructions. Bacterial DNA was quantified with a BioDrop analyser (Montreal Biotechnologies Inc., Canada). Microbiota composition was determined by 16S rRNA gene profiling (Génome Québec core service). Briefly, the V3-V4 region of the 16S rRNA gene was amplified using the primers 341F and 805R, followed by sequencing on an Illumina MiSeq platform (Illumina, CA, USA). The 16S primer sequences are shown in **Supplementary Table 1**. The resulting sequences were processed and analyzed using DADA2 package (v1.17) integrated in the R environment (available at http://www.R-project.org) to generate exact amplicon variants (ASV) for each sample from raw amplicon. Sequences were corrected for Illumina amplicon sequence errors, de-replicated, chimera removed, and merged for paired-end reads. Taxonomic assignment of reads was obtained using the RDP classification algorithm (v2.2) trained on the Silva reference database (Silva_v138). Relative abundance was performed to visualize the difference between conditions.

### Bacterial viability

Bacterial viability was measured exactly as described (Alonzo-De la Rosa et al., 2023). Aliquots of microbiota extracts (diluted 1:3 in PBS) were placed in 96-well plates and added with resazurin (0.15 mg/mL). Plates were incubated for 2 hours at room temperature before fluorescence measurements (550 nm excitation and 590 nm emission) using a hybrid multi-mode reader (Biotek).

### Schaeffer-Fulton endospore staining

Presence of spores was evaluated as previously described (Alonzo-De la Rosa et al., 2023). A droplet of microbiota was fixed on a glass slide by passing it over the flame. After placing a piece of paper towel on the slide, it was saturated with malachite green stain solution (0.5% w/v in water) and steamed for 5 minutes over a glass beaker containing boiling water to keep the paper towel moist. The paper towel was removed and the slides were washed with distilled water. Next, safranin stain solution (10 mL of 2.5% w/v in ethanol 95% and 90 mL of distilled water) was added and then washed with distilled water after 30 seconds. Images were taken with an inverted microscope.

### Nutritional content

In addition to acting in symbiosis, bacteria can serve as an energy source. Therefore, glucose, glycogen, glycerol and triglyceride contents were measured for all groups of microbiota extracts exactly as previously described (Alonzo-De la Rosa et al., 2023). Briefly, microbiota aliquots (30 μg DNA) were pelleted and mixed with either distilled water (for glucose and glycogen) or NP40 5% solution (for glycerol) and underwent 10 cycles of sonification with 50% amplitude (Branson Sonifier Cell Disruptor 185, LabX, USA). Triglycerides were extracted following the Folch method (Folch et al., 1957). Measurements were performed using kits following manufacturer’s instructions (glucose and glycogen: Abcam kit #ab65620/K646-100; glycerol: Abcam kit #ab65336/K622-100; triglycerides: Abcam kit # abcam 65336). Data were corrected for bacterial DNA content.

### *C. elegans* culture

*C. elegans* N2 wild-type strain was maintained and grown at 20°C on solid nematode growth media (NGM) plates seeded with 1 mL of *E.coli* OP50 cultures. OP50 was prepared in a liquid culture media (Terrific Broth Media) at 37°C with agitation overnight. Worms were synchronized using the alkaline hypochlorite method as described (Porta-de-la-Riva et al., 2012).

### Food choice

As described by our team (Alonzo-De la Rosa et al., 2023), an aliquot of 70 µL of microbiota (7 µg of bacterial DNA) of each group was placed at equal distance on peptone-free NGM plates with FUDR 50 μm. About 50 synchronized L4 worms were placed on the plates. Subsequently, the worms were located and counted under a microscope after 1, 24, 72, and 120 hours. The proportion of worms on food or not on food was calculated.

### Pharyngeal pumping

As previously described by our team (Alonzo-De la Rosa et al., 2023), using 24-well plates containing peptone-free NGM, microbiota (5 ng DNA) was added in the wells with one L4 synchronized worm. Pharyngeal pumping was counted for 1 min three times for each worm tested at days 1, 3, and 5 using an Olympus MVX10 microscope.

### Growth and fat mass accumulation

Body length and body size of 10-day adult worms were quantified using a Biosorter flow cytometer (Union Biometrica, MA, USA) using time of flight and extinction as proxy, respectively, as described (Pulak, 2006). Fat content was evaluated by Oil Red O staining in fixed worms exactly as described (Alonzo-De la Rosa et al., 2023). Briefly, worms were incubated overnight at RT in a 60% Oil Red O solution, prepared by diluting a 0.5% Oil Red O stock solution made in high-quality 100% isopropanol with double-distilled water. The next day, the worms were washed with M9 and resuspended in a 0.01% Triton solution with M9. Pictures were taken with an Olympus MVX10 microscope. Oil Red O staining intensity was quantified using the Image J software.

### Brood size

Using 24-well plates containing peptone-free NGM, microbiota (15 ng DNA) was added in the wells with one L4 synchronized worm as described previously by our team (Alonzo-De la Rosa et al., 2023). Each worm was transferred to a new well every day to count the eggs. Brood size was determined as the sum of total eggs laid by each individual worm.

### Quantification of bag of worms (Bag) and age-associated vulval integrity defect (Avid) phenotypes

Microbiota (10 g DNA) was added to peptone-free NGM plates with synchronized L4 worms and kept at 20°C as described (Alonzo-De la Rosa et al., 2023). Worms were transferred to new plates every day and the Bag and Avid worms were counted. The proportion of Bag and Avid worms were calculated.

### Lifespan

Equal quantities of each group of microbiota extracts were placed on NGM peptone free-plates with fluorodeoxyuridine (FUDR) 50 m, as described by our team (Alonzo-De la Rosa et al., 2023). Synchronized L4 worms were placed on the petri dishes (50 worms per plate) and kept at 20°C. The worms were transferred to a new plate every two days and the ones that did not respond to touch were considered dead. Maximum lifespan was recorded as the day at which the last worm died on a specific plate.

### Statistical analysis

Unless otherwise stated, all data are reported as means and standard error of the mean (SEM). One-way ANOVA was used to analyse nutritional content of microbiota extracts, body length, body size, brood size, Bag and Avid phenotypes, and maximal lifespan. Repeated measures ANOVA was used to analyze bacterial viability. Two-way ANOVA was used to analyze food choices and pharyngeal pumping. When applicable, data were further analyzed by Bonferroni’s *post-hoc* test.

## Results and discussion

### Long-term freezing of feces modifies microbiota composition but not bacterial viability of microbiota extracts

**Figure 1:**
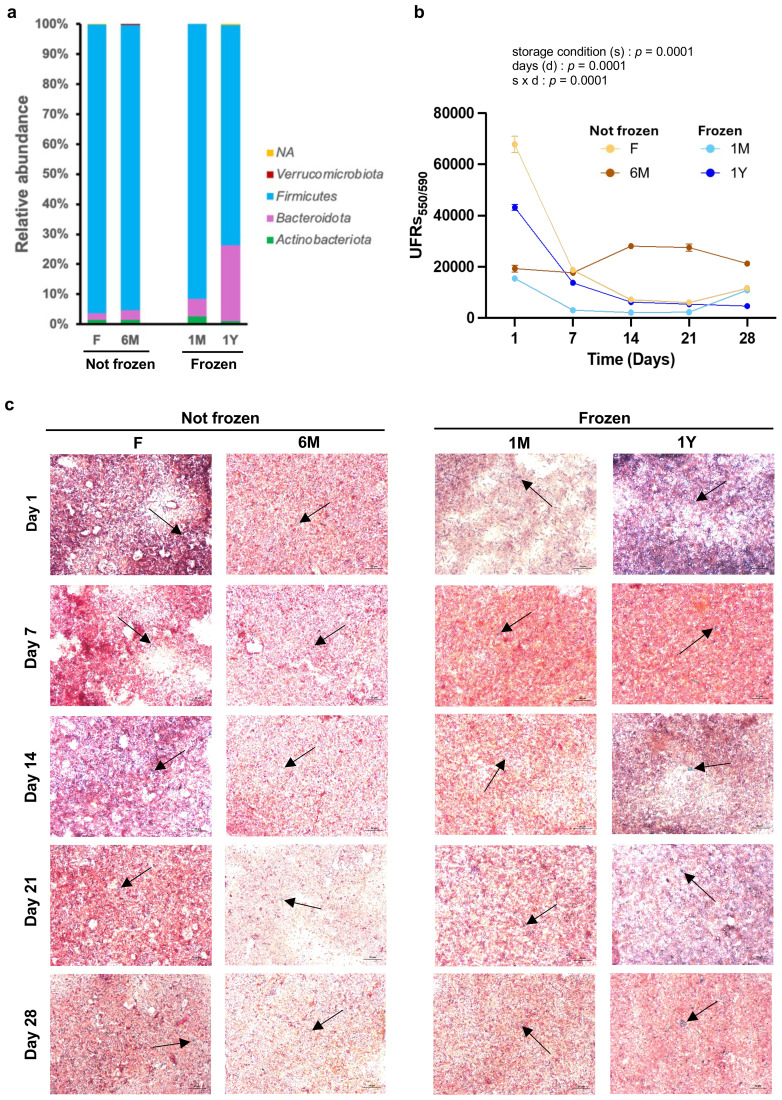
Long-term freezing of feces changes composition but does not modify bacterial viability of microbiota extracts. **(A)** Relative abundance expressed at the Genus level of bacteria from fecal microbiota extracts freshly used (F) or kept at room temperature for 6 months (6M) or as well as from extracts from thawed feces that have been previously frozen one month (1M) or one year (1Y). **(B)** Quantitative evaluation of bacterial viability assessed over 28 days in fecal microbiota extracts detailed in **(A)**. Each data point represents the relative fluorescence of three resazurin assays in duplicates. Values are mean ± SEM. Statistical comparisons by ANOVA repeated measures. **(C)** Representative images of Scheaffer-Fulton staining for each group of microbiota extracts at days 1, 7, 14, 21 and 28. Spores are stained by green (arrows) and bacterial vegetative cells are pink. Magnification is 63 x.

To test the impact of microbiota extracts on *C. elegans* biology according to their storage condition, feces from different animals were pooled into four distinct groups, namely those frozen for more than a year, those freshly harvested but frozen for a month, those freshly harvested and kept at room temperature, and those kept at room temperature for 6 months. We first evaluated microbiota composition by 16S rRNA sequencing. The combined results show that long-term freezing leads to robust changes in bacterial composition of the microbiota extracts, and surprisingly more than storing at RT for 6 months. Notably, storage at -80 °C increased the relative proportion of *Bacteroidota* at the expense of *Firmicutes*, and this effect was more pronounced after long-term freezing (**Figure 1A**). This contrasts with previous data obtained from human samples (Bahl et al., 2012; Dorsaz et al., 2020; Bilinski et al., 2022), and more studies in other cohorts of mice are required to test a possible species-specific impact of freezing. However, detailed analyzes between fresh and samples stored at room temperature for 6 months revealed interesting changes, with increased number of species from the *Atopostipes* genus and much lower number of species from the *Enterococcus* genus (**Supplementary Table 1**). *Atopostipes* are gram-positive, non-spore forming, facultative anaerobic bacteria, correlated with increases in inflammation (Khadka et al., 2023), whereas *Enterococcus* bacteria promote health benefits, with probiotic potential to assimilate total cholesterol (Nami et al., 2019) and positive healthspan effects in *C. elegans* (Sim et al., 2018). Taken together, these data clearly support that testing feces of similar period of storage time and temperature is crucial when comparing groups to avoid differences in microbiota composition.

Quantification of viability by resazurin assays revealed that microbiota extraction from feces (either fresh [F] or frozen for a month [1M] or a year [1Y]) caused a reduction of viability within one week to levels observed in microbiota extracts processed six months before [6M] (**Figure 1B**). This sharp decrease in bacterial viability in the first week after thawing and processing of feces may be due to the loss of strict anaerobic species, although this was not evaluated. However, consistent with previous findings (Alonzo-De la Rosa et al., 2023), viability of extracts remained relatively stable after that period for at least 28 days (**Figure 1B**), albeit with the limitation that bacterial content and community structure of these samples likely evolved during that time period. Nonetheless, all extracts showed spore formation for at least 28 days after processing (**Figure 1C**). For this reason, in this study, extracts from fresh or frozen feces were used for tests in worms between 7 and 28 days after processing and compared to extracts processed 6 months before and left at room temperature.

### Freezing does not alter nutritional content and preferences for microbiota extracts

**Figure 2:**
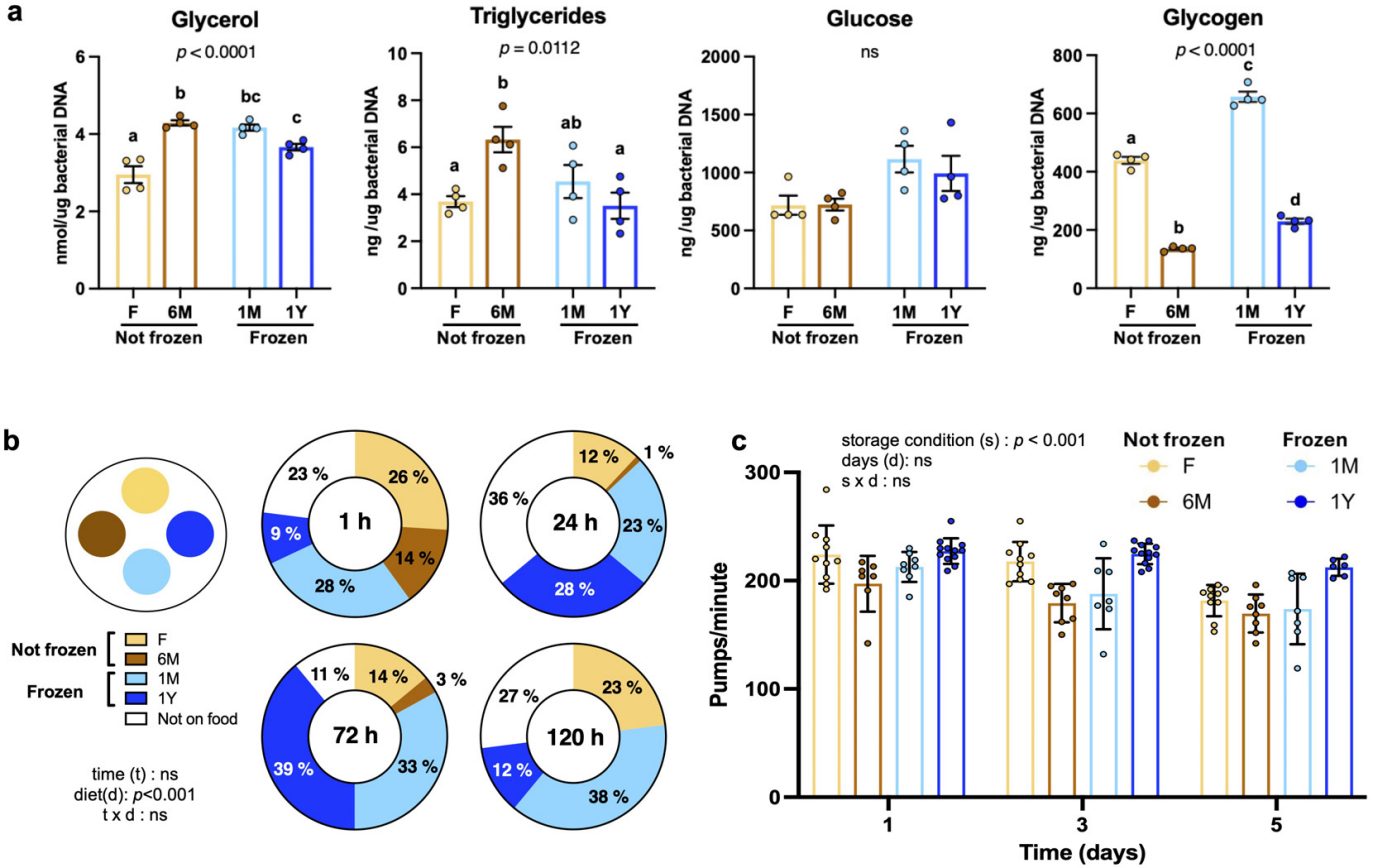
Freezing does not alter nutritional content and preferences for microbiota extracts. **(A)** Concentrations of glycerol, triglycerides, glucose and glycogen in microbiota extracts freshly used (F) or kept at room temperature for 6 months (6M) or as well as from extracts from thawed feces that have been previously frozen one month (1M) or one year (1Y). Data were corrected for bacterial DNA content. Bars are mean ± SEM of three measurements made in duplicates. Results analyzed by one-way ANOVA (*p* values shown on top of panels) followed by Bonferroni’s *post-hoc* test. Bars not sharing similar letters are significantly different from each other (*p* < 0.05). **(B)** Food preference assay between microbiota extracts described in **(A)**. Similar quantities of extracts were spotted on plates and synchronized worms were added. The number of worms crawling within extracts or not on food was counted after 1, 24, 72 and 120 hours. Data represent the percentage of worms for each condition and were analyzed by two-way ANOVA. **(C)** Pharyngeal pumping per minute in worms fed with the microbiota extracts described in **(A)** for one, three and five days. Each point represents one worm. Bars are mean ± SEM and were analyzed by two-way ANOVA.

Since worms use microbiota not only for symbiotic relationships but also as a food source, the nutritional content of microbiota extracts was quantified. Microbiota readily extracted from fresh feces contained slightly less glycerol than other groups, whereas triglyceride levels were slightly higher in non-frozen 6M samples compared to other extracts (**Figure 2A**). Glucose content was similar between groups (**Figure 2A**). Interestingly, long-term storage, either at room temperature or -80 °C, was associated with a lower glycogen content (**Figure 2A**). All values were within range observed previously (Alonzo-De la Rosa et al., 2023).

When placed in contact with microbiota, worms rapidly and robustly avoided the non-frozen 6M group compared to other extracts (**Figure 2B**). This effect lasted for at least 120 hours. No statistical difference in food preference was observed between the three other groups (**Figure 2B**). Not only did worms not prefered non-frozen 6M microbiota when facing a choice, they pumped food at a lower rate when placed on non-frozen 6M extracts as their only food option (**Figure 2C**). These findings strongly suggest that freezing of feces does not alter feeding preferences or pumping activity, but that processing of extracts and storing at room temperature for 6 months before using in experiments leads to avoidance.

### Freezing does not impede growth and fat accumulation in worms fed microbiota extracts

**Figure 3:**
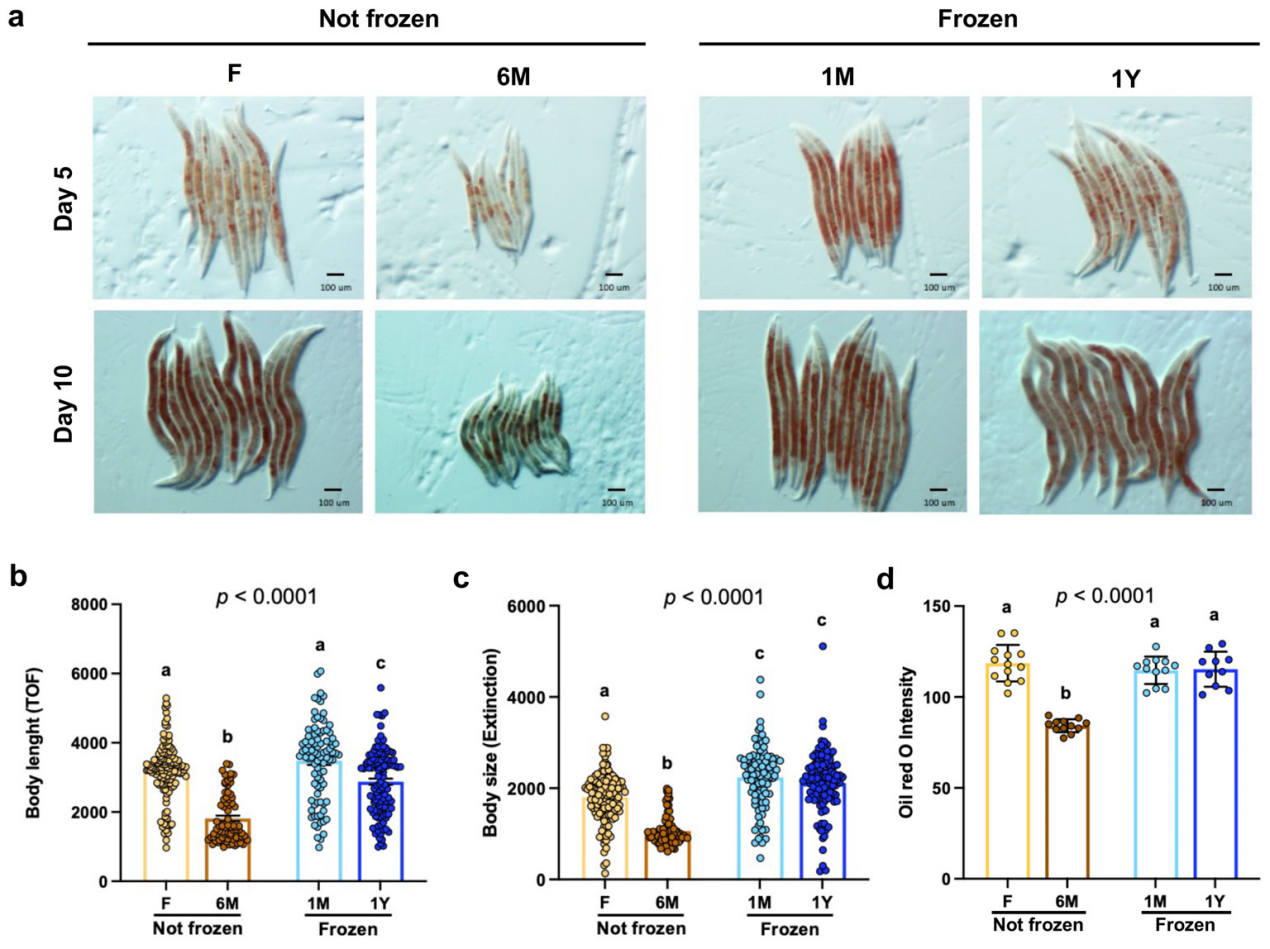
Freezing does not impede development and fat accumulation in worms fed microbiota extracts. **(A)** Representative images of Oil Red O staining of adult worms fed for 5 or 10 days with extracts freshly used (F) or kept at room temperature for 6 months (6M) or as well as from extracts from thawed feces that have been previously frozen one month (1M) or one year (1Y) detailed in **(A)**. Magnification is 50 x. Bars indicate 100 µm. **(B)** Body length of synchronized 10-day adult worms fed 10 days with microbiota extracts detailed in **(A)**. Each point represents one worm. Bars are mean ± SEM. Results analyzed by one-way ANOVA (*p* values shown on top of panels) followed by Bonferroni’s *post-hoc* test. Bars not sharing similar letters are significantly different from each other (*p* < 0.05). **(C)** Body size of worms described in **(B)**. Data analyzed as in **(B)**. **(D)** Quantification of Oil Red O staining intensity of worms fed for 10 days as detailed in **(A)**. Data analyzed as in **(B)**.

Next, the impacts of different feces storage conditions on *C. elegans* biology were tested. As clearly evidenced by light microscopy (**Figure 3A**) and FACS analyses (**Figures 3B, C**), body length (**Figure 3B**) and body size (**Figure 3C**) were significantly lower in worms fed with non-frozen 6M extracts compared to worms fed with the other extracts. However, worms fed with extracts from frozen feces displayed similar growth than those fed with freshly extracted samples (**Figures 3A–C**). Consistently, body fat content, as indexed by Oil Red O staining (**Figure 3A**), was also significantly lower in worms fed with non-frozen 6M extracts compared to worms fed with the other extracts (**Figure 3D**). These data suggest that lower preference and intake of microbiota extracts processed 6 months before experiments and conserved at room temperature have strong deleterious effects on development and fat accumulation, whereas those from frozen feces do not.

### Freezing does not hinder reproduction and maximal lifespan in worms fed microbiota extracts

**Figure 4:**
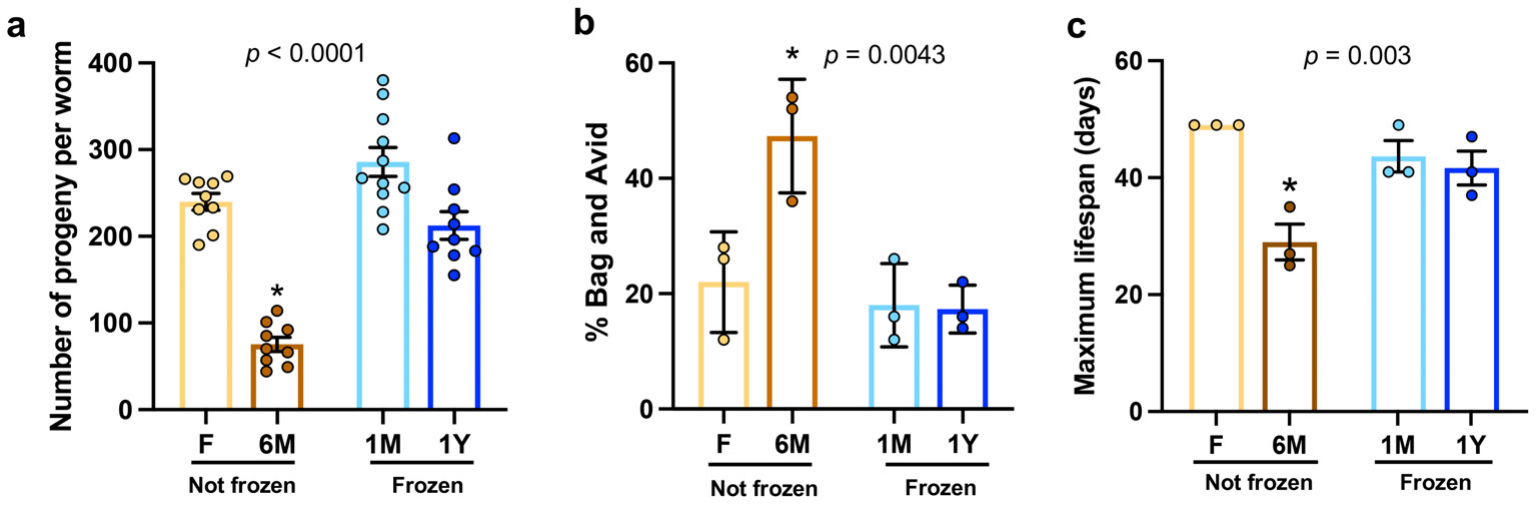
Freezing does not hinder reproduction and maximal lifespan in worms fed microbiota extracts. **(A)** Brood size per worm in groups fed with microbiota extracts freshly used (F) or kept at room temperature for 6 months (6M) or as well as from extracts from thawed feces that have been previously frozen one month (1M) or one year (1Y). Each point represents one worm. **(B)** Percentage of worms showing a Bag and an Avid phenotype as quantified over the reproductive period. Three plates per groups, 50 worms per plate were evaluated. **(C)** Maximal lifespan recorded in plates of synchronized worms described in **(A)**. Three plates per groups, 50 worms per plate were evaluated. Each point represents one plate. In each panel, bars are mean ± SEM. Results analyzed by one-way ANOVA (*p* values shown on top of panels) followed by Bonferroni’s *post-hoc* test. Asterisks represent statistical differences compared to other groups (*p* < 0.05).

It is well established that food intake and nutritional content have profound impacts on *C. elegans* development, reproduction, and longevity (Pang and Curran, 2014; Collins et al., 2016; Gelino et al., 2016; Hacariz et al., 2021). In line with this and consistent with the findings described above, worms fed with non-frozen 6M extracts showed significantly and substantially reduced brood size (**Figure 4A**), higher proportions of Bag and Avid phenotypes (**Figure 4B**), and lower maximal lifespan (**Figure 4C**) compared to worms grown on other microbiota extracts. In contrast, no difference was observed between worms fed with microbiota from frozen feces and those with freshly processed samples (**Figures 4A–C**). These results demonstrate that freezing of feces before microbiota extraction and testing in worms does not negatively impact development, reproduction, and maximal lifespan, whereas using samples extracted and left at room temperature after a long period of time is detrimental for normal healthspan in *C. elegans*.

In conclusion, the findings described in this methodology study may have several impacts, the main one being that using frozen feces to test for impacts of microbiota in *C. elegans* appears possible without affecting normal biology and healthspan, which supports previous data on fecal transplantation in other models (Costello et al., 2015; Bilinski et al., 2022). This would allow the use of already existing stool samples stored in biobanks for high-throughput phenotype screenings in worms (assuming that freezing time is similar between tested samples) without the need for repeating protocols in mice to obtain novel feces, which would reduce cost, time, and the number of experimental animals used. However, our results also demonstrate that long-term storage of microbiota extracts at room temperature increases subsequent avoidance behaviors in worms, leading to lower energy intake that robustly impacts on nematode development and reproduction. The deleterious effects of the 6M extracts could be due to changes (content and/or activity) of specific bacterial species triggering the production of several metabolites and formation of reactive oxidative species (Feng et al., 2023). In this regard, the slightly higher resazurin activity in the 6M group (**Figure 1B**) is consistent with a potentially higher global redox state that could have had a subsequent altering effect on *C. elegans* biology (Seixas et al., 2021). These possibilities need to be thoroughly evaluated.

In addition, a strong limitation of the study is the change in bacterial composition that occurs during freezing (Dorsaz et al., 2020) as well as a likely loss of strictly anaerobic species during the extraction process, which impedes their study and lowers the extent of potential conclusions, especially if these species strongly contribute to palatability or symbiosis. Nevertheless, given the crucial need to analyze the complexity of host-microbiota interactions with all possible combinations, the present strategy employing existing frozen feces in worms offers timely and cost-efficient arguments before designing larger experiments in mammalian models.

## Data Availability

The raw data supporting the conclusions of this article will be made available by the authors, without undue reservation.
