# Supplementary material for: Effects of feces storage conditions for host-microbiota screenings in C. elegans
Source: Front Microbiomes. 2024 Dec 19;3:1426254. doi: 10.3389/frmbi.2024.1426254 (PMC12993547; doi:10.3389/frmbi.2024.1426254)
Supplement: Supplementary file 2 [file Table1.docx]

**Supplementary Table 1**. Primer sequences for 16S sequencing.

| 341F | 5′CCTACGGGNGGCWGCAG-3′ |
| --- | --- |
| 805F | 5′GACTACHVGGGTATCTAATCC-3′ |

**Supplementary Table 2**. Counts of bacterial families of the five bacteria genus that are the most different between fresh and 6M microbiota extracts after 16S sequencing. Table pertaining to Fig. 1A.

|  | **Not frozen** | | **Frozen** | |
| --- | --- | --- | --- | --- |
| **Genus** | **Fresh** | **6M** | **1M** | **1Y** |
| *Adlercreutzia* | 0 | 63 | 176 | 0 |
| *Atopostipes* | 661 | 1634 | 544 | 34 |
| *Enterococcus* | 1173 | 420 | 5579 | 7212 |
| *Sporosarcina* | 2903 | 667 | 146 | 81 |
| *Lachnospiraceae NK4A136 group* | 378 | 1051 | 732 | 45 |
